# Herpesviruses Placating the Unwilling Host: Manipulation of the MHC Class II Antigen Presentation Pathway

**DOI:** 10.3390/v4081335

**Published:** 2012-08-22

**Authors:** Jianmin Zuo, Martin Rowe

**Affiliations:** Cancer Research UK Birmingham Cancer Centre, University of Birmingham, Birmingham B15 2TT, UK; Email: m.rowe@bham.ac.uk

**Keywords:** herpes viruses, MHC class II, CD4 T cell, immune evasion

## Abstract

Lifelong persistent infection by herpesviruses depends on the balance between host immune responses and viral immune evasion. CD4 T cells responding to antigens presented on major histocompatibility complex class II (MHC-II) molecules are known to play an important role in controlling herpesvirus infections. Here we review, with emphasis on human herpesvirus infections, the strategies evolved to evade CD4 T cell immunity. These viruses target multiple points on the MHC class II antigen presentation pathway. The mechanisms include: suppression of CIITA to inhibit the synthesis of MHC class II molecules, diversion or degradation of HLA-DR molecules during membrane transport, and direct targeting of the invariant chain chaperone of HLA-DR.

## 1. Introduction

Members of the family herpesviridae are large DNA viruses that commonly cause disease in animals. In humans, eight distinct viruses have been identified (Table 1), with representation in each of the three subfamilies: alpha-herpesviruses, beta-herpesviruses and gamma-herpesviruses [1]. Most of the human population carry one or more herpesviruses, for example Epstein-Barr virus (EBV) has colonized more than 90% of the adult human population worldwide.

Primary infection with herpesviruses may be clinically silent or manifest as acute disease that is usually followed by establishment of lifelong latent persistence with occasional reactivation. A key component of the virus-host balance during persistent infection is the immune response elicited by the host, which includes CD8 and CD4 T cell responses targeting a very broad range of virus antigens [2]. In immunocompromised patients, the herpesviruses can be reactivated with a resultant predisposition to viral diseases due to the loss of immune control [3,4,5]. Whilst the potent cellular immune responses normally restrict the pathogenic potential of herpesviruses, it is evident that they fail to eliminate the virus completely from its host. This implies that herpesviruses have very effective immune evasion mechanisms. Strategies for immune-evasion may be broadly defined as passive or active. Passive evasion is exemplified by latency, or restricted expression of viral target antigens, rendering the infected cells invisible or less visible to the immune system [6]. Active evasion involves viral gene products (immunevasins) that interfere with specific immune functions such as antigen presentation; one of the earliest reported mechanisms of active evasion by a human herpesvirus was that of HSV-encoded ICP47 interacting with and inhibiting the TAP transporter of antigen processing [7,8,9]. It is important to recognise that immune evasion mechanisms are not absolute, but rather they serve to blunt the effectiveness of immune responses. In the healthy infected host the establishment of an equilibrium between host immune responses and viral evasion mechanisms allows virus persistence with minimum pathogenic consequences. 

**Table 1:** Herpes viruses and their associated diseases.

Subfamily	Virus name	Nomenclature	Associated diseases
Alpha-herpesviruses	Herpes Simplex-1	HSV-1 or HHV-1	Gingivostomatosis, Cold sores, Encephalitis
	Herpes Simplex-2	HSV-2 or HHV-3	Genital herpes, Cutaneous herpes, Encephalitis, Meningoencephalitis
	Varicella zoster virus	VZV or HHV-3	Chickenpox, Shingles
Beta-herpesviruses	Cytomegalovirus	HCMV or HHV-5	Mononucleosis, Hepatititis, Pneumonitis
	Human herpesvirus-6	HHV-6	Exanthum subitum, Mild febrile illness
	Human herpesvirus-7	HHV-7	Exanthum subitum, Mild febrile illness
Gamma-herpesviruses	Epstein-Barr virus	EBV or HHV-4	Mononucleosis, Burkitt’s lymphoma, post-transplant lymphoproliferative syndrome (PTLD), nasopharyngeal carcinoma,
	Kaposi’s sarcoma-associated herpesvirus	KSHV or HHV-8	Kaposi’s sarcoma, primary effusion lymphoma, some types of multicentric Castleman’s disease

The identification and functional characterisation of immunevasins of herpesviruses targeting the major histocompatibility complex class I (MHC-I) antigen presentation pathways has been extensively studied (for reviews, see [10,11,12,13,14]). A picture emerges that each member of the herpesviridae family encodes multiple immunoevasins which together target multiple steps of the MHC-I antigen presentation pathway. Less well understood, however, are the mechanisms evolved by herpesviruses to manipulate the major histocompatibility complex class II(MHC-II) antigen presentation pathways. In this review, we will outline and discuss recent developments in the research on the interference by herpesvirus immunevasins with MHC-II antigen presentation pathways to CD4 T cells.

## 2. Why MHC-II Evasion Is Important

Historically cytotoxic CD8 T cells have been considered the crucial immune effector cells to mediate pathogen clearance by killing the infected host cells. Indeed, the success of adoptive T cell therapy for EBV-associated lymphoproliferations in transplant patients [15,16] lended support for this view. The role of CD4 T cells in anti-viral immunity was for some time considered as mostly indirect by providing help to promote the generation and functions of B cells and CD8 T cells. However, evidence is accumulating that many CD4 T cells have cytotoxic functions and other direct antiviral roles [17]. This puts a different perspective on the significance of the finding that the presence of CD4 cells admixed with CD8 cells in the immune T cell infusions used in adoptive immunotherapy of transplant patients correlates with improved treatment outcome [18].

In animal models of herpesvirus infections, there is strong evidence for CD4 immune T cells affording anti-viral protection independently of their traditional helper activities. For example, in the case of the MHV68 gamma-herpesvirus mouse model, Stevenson *et al*. used antibody treatment to deplete different T cell subsets to show that the mice can survive MHV68 infection even when the CD8 T cell population was greatly diminished, whereas the concurrent removal of both CD4 and CD8 T cell subsets proved invariably fatal [19]. In a separate study, Christensen *et al*. showed that CD4 T cells can be directly antiviral, independently of CD8 T cells or B cells, in MHV68-infected mice [20]. In a more recent study by Sparks-Thissen *et al*. using T-cell receptor (TCR) transgenic mice that had CD4 T cells specific for OVA were challenged with the a recombinant MHV68 expressing OVA [21], the OVA-specific CD4 T cells were found to limit the acute MHV68 replication and prolonged the life of transgenic mice. It was subsequently shown by Stuller *et al*. that CD4 T cells mediate anti-viral control by two independent mechanisms, IFN-γ production and cytotoxicity [22]. Altogether, these data showed that CD4 T cells can control replication, prevent lethal infection, and inhibit the establishment of latency in MHV68 infection. Similarly, in mouse models for HSV infection, immune CD4 T cells play an important role in clearance of infectious virus at neural sites following HSV-1 infection [23], and can protect mice from lethal infection by HSV-2 when adoptively transferred CD4 T cells expressed functional FasL that induces apoptosis of Fas-expressing target cells *in vitro* [24]. CD4 T cell mediated protection in the absence of CD8 T cells and B cells has also been described in animal models of infection by VZV [25] and in infections by other virus families, including influenza [26], poliovirus [27] and West Nile virus [28]. 

In addition to causing acute and chronic infections, the human gamma-herpesviruses EBV and KSHV could be oncogenic. Cell growth transformation of human B cells by EBV is achieved with remarkable efficiency *in vitro*, but can be abrogated by the EBV-specific immune T cell memory present in the peripheral blood of EBV-positive donors. Whilst there is a large body of evidence pointing to the role of cytotoxic CD8 T cells in the inhibition of outgrowth of transformed B cells in this model [29], depletion experiments also revealed a role for CD4 T cells [30]. Subsequent studies showed that outgrowth of EBV transformed B cells could also be mediated by EBV-specific CD4 T cells primed *in vitro* by dendritic cells (DCs) from EBV sero-negative donors [31], or by EBNA1-, EBNA2-, LMP1- or LMP2-specific CD4 T cell clones generated from EBV sero-positive donors [32,33,34]. Furthermore, taking the advantage of the animal model of MHV-68 oncogenic gamma-herpesvirus, Robertson *et al*.showed that MHV68-specific CD4 T cells, but not CD8 T cells can eliminate the tumors that were induced by injection of a MHV68-infected B cell lymphoma cell line into T cell–deficient (nude) mice [35]. Taken together, these data showed that virus-specific CD4 T cell responses can control gamma-herpesvirus induced lymphocyte growth-transformation independently of CD8 T cells. 

### 2.1. Herpesvirus Infections Generate Broad Range Anti-Viral CD4 T Cell Responses

The breadth and specificity of herpesvirus-specific CD8 and CD4 T cell responses has been extensively studied more than a decade. Below, we summarise the CD4 T cell responses to human herpesvirus infections. 

#### 2.1.1. HSV

Although there were a few prior reports about the CD4 T cell responses to HSV [36,37], a recent systematic and genome-wide scan by Koelle’s group [38] provided a more complete picture of the repertoire of CD4 T cell responses. In this latter study HSV-1 specific CD4 memory T cells were reactivated with UV-killed cell-associated HSV-1. Deploying a near complete collection of HSV-1 ORF clones to identify the specificity of the polyclonal virus-specific CD4 T cells, it was shown that the average number of HSV-1 open reading frames (ORFs) recognized per individual was 22.8 ± 7.0 (mean ± SD), and 74 unique polypeptide antigen targets were identified. On a population basis, the most prevalent CD4 responses were envelope glycoproteins gB1 and gD1, and tegument protein VP11/12, encoded by the UL46 gene [38].

#### 2.1.2. HCMV

Sylwester *et al*. carried out an inclusive screening of CD4 and CD8 T cell responses in 33 HCMV seropositive donors, with 13,687 overlapping 15 mer peptides covering all 213 known or predicted HCMV ORFs [39]. With regards to CD4 T cell responses, 5 ORFs (UL55, UL83, UL86, UL99, and UL122) were recognized by more than half of the studied subjects, and 40 ORFs were recognized by CD4 T cells in at least 4/33 subjects. The immunogenic ORFs span all temporal and functional categories. CMV specific responses often accounted for a remarkably high proportion of the overall CD4 peripheral blood T cell population, with 10/33 seropositive subjects displaying total HCMV-specific CD4 T cell responses that represented ≥20% of their circulating memory repertoire.

#### 2.1.3. EBV

Because of the available cell models, T cell responses to EBV have been extensively studied for target antigens expressed in ‘latent’ growth-transformed cells as well as those in ‘lytic’ virus-producing cells. In EBV latency, nine viral antigens are expressed, including six Nuclear Antigens (EBNAs), a vBCL2 homologue (BHRF1) and two Latent Membrane Proteins (LMP1 and LMP2). CD4 T cell responses are broadly targeted across all nine proteins, although up to half of the currently defined CD4 epitopes (but not necessarily those eliciting the strongest responses) derive from EBNA1 [2]. Many of these CD4 T cells recognize and inhibit the outgrowth of EBV-transformed normal B cells (lymphoblastoid cell lines, LCLs) *in vitro*, and some have cytotoxic function enabling them to directly kill LCLs or EBV-positive tumour cell lines such as Burkitt lymphoma lines [34,40,41,42,43]. 

Whilst EBV-transformed normal LCLs display a predominantly non-productive ‘latent’ infection, most such lines contain a minor subpopulation of cells that have spontaneously switched into the lytic cycle with sequential expression of two immediate early (IE) genes, about 30 early (E) genes, and about 30 late (L) lytic genes. It has long been recognised that some EBV lytic cycle antigens are strongly immunogenic for CD4 T cell responses [44,45]. More recently, Long *et al*. undertook a more systematic analysis of CD4 T cell responses to eight lytic proteins in 14 virus-immune donors [46], showing that the CD4 T cell response is widely distributed across IE, E, and L antigen targets. Remarkably, all the lytic antigen-specific CD4 clones tested had cytotoxic function, with target cell killing being associated with cell surface mobilization of CD107a [46].

#### 2.1.4. KSHV

There are few reports describing immune T cell responses to KSHV. Recently, Sabbah *et al*.investigated the magnitude and the specificity of CD4 T cell responses to 4 of the latent antigens that are expressed in KSHV-associated primary effusion lymphoma (PEL); LANA, vFLIP, cCyclin, and Kaposin [47]. Generally, responses in healthy infected donors to these antigens were very weak compared to what is observed with responses to the closely related EBV gamma-herpesvirus, and the majority of CD4 T cell clones generated were specific for LANA derived peptides. 

#### 2.1.5. VZV

VZV-specific CD4 T cells can be detected during acute VZV infection. Several studies have shown that VZV-specific CD4 T cells recognize ORF4 [48], glycoprotein I [49], and ORF63 [50]. Furthermore, it appears that VZV-specific CD4 T cells circulate at much higher frequencies compared to VZV-specific CD8^+^ T cells [49,51]. 

#### 2.1.6. HHV-6

CD4 T cells responding to HHV-6 were observed at frequencies below 0.1% of total T cells, and they can release IFN-γ and IL-10 after stimulation [52]. 

### 2.2. Target Cells Expressing MHC-II and Presenting Peptide

Normally, MHC class II expression is largely restricted to professional antigen-presenting cells (APCs), which potentially limits the protective potential of virus-specific cytotoxic CD4 T cells. However, MHC-II expressing professional APCs are natural targets for many herpesviruses; for example, B cells are target cells for EBV, KSHV. Furthermore, inflammatory cytokines such as IFN-gamma that are induced by virus infection can upregulate MHC-II expression on epithelial and endothelial cells, which are targets for all herpesviruses, enabling them to present antigen to CD4 T cells. 

**Figure 1:**
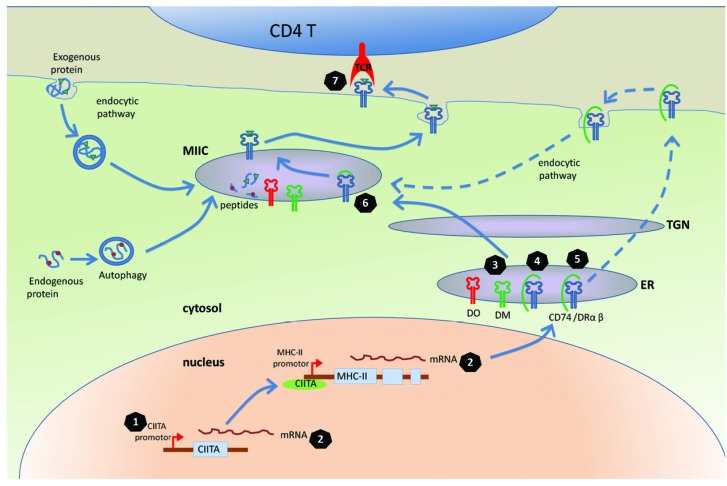
The modulation of MHC-II antigen presentation by herpesviruses.Simplified schematic showing the pathways of antigen processing and presentation via MHC-II complexes within a target cell (green) for recognition by an immune CD4 T cell (blue). Multiple points of modulation by viral gene products are indicated: (**1**) Interference with CIITA transcription: EBV BZLF1 and KSHV vIRF3 inhibit the CIITA promoter and subsequently inhibit the transcription of MHC-II molecules. (**2**) Degradation of mRNA: HSV-1 UL41, EBV BGLF5 and KSHV SOX can degrade the mRNA of CIITA and MHC-II molecules. (**3** and **4**) Manipulation of HLA-DM and DR in ER: HCMV US2, US3, pp65 and HSV-1 gB can associate with DM and/or DR and impair the loading and presentation of MHC-II peptides. (**5**) Manipulation of CD74: EBV BZLF1 can modulate CD74 post-translationally, interfering with the normal transport and loading of peptides to MHC-II molecules. (**6**) Diversion to exosomes: HSV-1 gB associates with DR after CD74 is released from DR, reducing the surface expression of DR. (**7**) Manipulation of T cell receptor (TCR) recognition: EBV gp42 can abolish the interactions between TCR on the CD4 T cell with MHC-II peptide complexes.

There are two distinct pathways, endogenous and exogenous, by which antigen may be processed to presentation via MHC complexes. For MHC-II, most antigens are presented through an exogenous pathway; secreted antigen, antigen from dead cells, and released virions may be taken up by neighbouring cells by various mechanisms then processed via lysosomal vesicles to bind MHC-II complexes (see Figure 1). Alternatively, some endogenously synthesised antigens may be processed within the cell. With regards to the well-characterised EBV latent antigen targets, most (for example, EBNA2, EBNA3B, EBNA3C, BHRF1) are processed to MHC-II via exogenous processing [53,54,55], while EBNA1 is exceptional in being processed only via endogenous processing mechanisms which include macroautophagy [56,57]. In addition to the autophagy pathway, endogenous viral antigen (for example, HCMV encoded gB) may be processed through trafficking to endosomal compartments and processing by acidic proteases [58]. 

## 3. Mechanisms for Interfering with the MHC-II Antigen Presentation Pathway

Effector CD4 T cells can be generated during both chronic and acute infection with herpesviruses infection, and are important for controlling the infection through both cytotoxic and cytokine-dependent mechanisms. How then do these viruses modulate MHC-II antigen presentation pathways to avoid complete elimination and to enable them to establish a successful persistent infection?

### 3.1. The MHC-II Antigen Presentation Is Inhibited in the Cells Infected with Herpesviruses

Evidence has accumulated over the past dozen years or so to support the idea that modulation of MHC-II presentation is a common feature of all human herpesviruses [59]. In one early report, analysis of the distribution of MHC-II expressing cells in skin biopsies taken from individuals with acute varicella or herpes zoster showed that MHC-II DR-α transcripts were detected in cells in proximity to virus-infected cells but were never detected in the virus-infected cells themselves [60]. An *in vitro* investigation of HCMV infection of the U373-CIITA cell line revealed a virus-mediated downregulation of the cell surface MHC-II expression in the absence of any significant change in the levels of total cell steady-state MHC-II protein or mRNA [61]. In this study, it was observed that MHC-II positive vesicles were retained in an abnormal perinuclear location. 

Importantly, other studies have used functional T cell assays as readout to investigate the biological consequences of the modulation of MHC-II expression. Thus, HSV-1 infection of LCL target cells substantially reduced their potency as stimulators of antigen-specific CD4 T cell proliferation and cytokine release [62]. More recently, we have shown that the MHC-II antigen presentation is impaired in LCLs entering EBV lytic cycle [63]. 

### 3.2. Molecular Mechanisms of Herpesviruses’ Modulation of MHC-II Antigen Presentation

To understand the molecular mechanisms by which herpesviruses modulate MHC-II antigen presentation pathway, we will first briefly summarise selected aspects of the process of MHC-II antigen presentation (Figure 1) that has been reviewed in more detail elsewhere [64,65]. MHC-II molecules are normally co-ordinately expressed under the regulation of the master regulator of transcription, CIITA. The MHC-II α-chain, β-chain and invariant chain (Ii; also known as CD74) are synthesized and assembled in the endoplasmic reticulum (ER), where the association of CD74 with the αβ dimer prevents premature binding of peptides. A cytosolic di-leucine-targeting motif of CD74 directs MHC-II complexes to the endocytic pathway, either directly from the trans-Golgi network or via rapid internalization from the cell surface. Proteases within a special endosome called the MHC II compartment cleave the CD74 to enable peptide binding within the HLA class II peptide-binding groove. This process requires the involvement of chaperones, HLA-DM and HLA-DO. Stable MHC-II/peptide complexes are then presented on the cell surface, where they can be recognized by CD4 T cells. 

#### 3.2.1. Targeting CIITA, the Master Regulator of MHC Class II Gene Expression

CIITA is a transcriptional co-activator that lacks intrinsic DNA-binding function. It enhances transcription of MHC class II genes through interacting with transcription regulatory proteins, forming a stable enhanceosome that can bind to the regulatory module of the promoter of MHC-II molecules [66]. CIITA would therefore appear to be a vulnerable target for viral modulation of MHC-II gene expression. 

Using the EBV positive tumour cell line Raji, Li *et al*. demonstrated that the EBV-encoded IE protein, BZLF1, which is the master regulator of EBV lytic cycle, can bind to the CIITA promoter and strongly inhibit the transcription and constitutive expression of CIITA molecules [67]. This inhibition of CIITA, in turn can downregulate MHC-II DR expression. Another herpes virus protein vIRF3 from KSHV, which is expressed in latently infected primary effusion lymphoma (PEL) cells also inhibits the transcription of CIITA and suppresses the expression of MHC-II molecules [68]. In that study knockdown of vIRF-3 in KSHV-positive PEL cell lines using small interfering RNA (siRNA) technology resulted in increased MHC II levels. By using a more detailed luciferase reporter assay, the inhibition by vIRF3 was mapped to the IFN-γ responsive CIITA promoters, PIV and PIII. 

MHC-II molecules are constitutively expressed on professional APC cells, but the expression can be induced on other cells by IFN-γ through the induced transcription of CIITA [66]. In this context, HCMV infection of U373 MG cells caused a 13.5-fold reduction in the level of CIITA transcripts induced following treatment with IFN-γ, with a concordant inhibition of induced HLA-DR synthesis [69]. This inhibition of CIITA was traced to a defect downstream of IFN-γ induced STAT1 phosphorylation and nuclear translocation, although the viral gene responsible was not identified. It was also shown that the suppression of DR synthesis inhibits recognition by CD4 T cells specific for the major immediate-early protein, IE1. Importantly, these effects of HCMV were abrogated when the U373 MG target cells were transfected with a CIITA expression plasmid prior to infection with HCMV. Similarly to that observed with HCMV, VZV infection of human foreskin fibroblasts can prevent the IFN-γ induced CIITA expression, and consequently inhibit the transcription and expression of MHC-II molecules [60]. 

#### 3.2.2. Targeting Transport of DR

Correct transport of DR is essential for the MHC-II molecules to present the right peptides. The first reported identification of a viral inhibitor of the MHC-II pathway was the HCMV-encoded protein US2, which causes the degradation of MHC-I [70] and also of DR-α and DM-α, two essential proteins in the MHC-II antigen presentation pathway [71]. The latter study by Johnson’s group used an inducible replication-defective adenovirus vector expressing US2 to infect and induce the US2 expression in U373-CIITA cells. By radiolabeling and immunoprecipitation experiments, US2 was shown to bind MHC-II molecules, including DR-α, DR-β and CD74. A rapid loss of the MHC-II molecules, especially DR-α and DM-α, was observed with US2 expression, which could be reversed by proteasome inhibitors. It was hypothesized that the US2 glycoprotein can recognize the shared protein structures but not amino-acid sequence in these distinct MHC-II molecules. Finally, by using functional CD4 T cells, they showed that expression of US2 reduced the ability of cells to present antigen to CD4 T cells. Subsequent studies showed that the C-terminal domain of US2 plays an essential role in the degradation process [72,73]. Using *in vitro* assay based on recombinant protein, Gewurz *et al*. could not confirm the association of US2 with DR and DM, raising the possibility that the mechanism of US2 action is cell-type dependent [74]. 

The HCMV genome region encompassing US2-US11 region encodes four homologous glycoproteins, US2, US3, US6, and US11, all of which can modulate the MHC-I antigen presentation pathway. Johnson’s group that first reported US2 as MHC-II evasion protein also subsequently examined seven glycoproteins of the US2-US11 gene cassette for possible effects on the MHC-II antigen presentation pathway, using replication-defective adenovirus vectors and functional CD4 T cell assays. In addition to US2, they found that US3 also can inhibit recognition of target cells by CD4 T cells [75]. By radiolabeling and immunoprecipitation methods, US3 was found to not affect the synthesis, stability, nor Golgi transport of MHC-II proteins but could bind to MHC-II DR αβ complexes in the ER and reduce their binding with CD74. So in the US3 expressing cells, the MHC-II αβ complexes can move normally from the ER to the Golgi, but were not sorted efficiently to the MHC-II loading compartment. As a consequence, formation of peptide-loaded MHC-II complexes was reduced. It was postulated that by acting through different molecular mechanisms, US2 and US3 may cooperate in the context of natural HCMV infection to inhibit MHC-II mediated presentation of viral antigen to CD4 T cells.

Söderberg-Nauclér’s group independently demonstrated that HCMV-infected macrophages also exhibited a reduced expression of MHC-II molecules, which was mediated by 2 different mechanisms, at an early (1 day after infection) and at a late (4 days after infection) time point after infection [76]. Infection with a mutant HCMV-RV670, that is deleted for US1-9 and US11, substantially impaired the late effect; implicating a role for one or several genes in the US2-US11 region. The early effect on MHC-II expression was retained by UV-inactivated virus and by the deletion mutant HCMV, suggesting that a virion component may be responsible. One possible candidate is pp65, as the surface expression of HLA-DR was not reduced on cells infected with RVAD65, a mutant lacking pp65 protein [77]. HCMV infection of cells usually results in accumulation and degradation of HLA-DR in vacuoles or lysosomes near the nucleus, but this phenomenon was not observed in cells infected with the pp65-deficient mutant virus. 

The binding of viral gene products to DR molecules and subsequent perturbation of transport is not restricted to HCMV. Using a bioinformatics approach, Sievers *et al*. identified a sequence in gB from HSV-1 (strain 17) that is identical to the highly conserved HLA-DR binding motif in human CD74 [78]. Confirmation of the significance of this sequence identity was provided experimentally by the physical association of HSV-1 gB with three MHC-II DR allotypes, including DR1, DR3 and DR4. 

Using the HSV-1 infected B cell model, the Koch group showed that HSV-1 not only can bind to DR but also can bind to DM independently of the DR expression [79]. Furthermore, they showed that HSV-1-encoded gB can compete with CD74 for binding to MHC-II heterodimers. Both gB-associated DR and DM heterodimers can be exported from the ER to Golgi compartments. But the association of DR with HSV-1 gB changes the intracellular localization and hence reduces the DR expression on the cell surface. More recent work from the same group showed that the gB/DR complexes are resistant to Endo H treatment and are free of invariant chain, CD74. This leads to another hypothesis that HSV-1 gB associates with DR not in the ER, but rather after CD74 is released from DR [80]. The same study also showed that DR and gB are contained in morphologically altered endocytic vesicles containing the late endosomal marker CD63. Furthermore, the gB/DR complexes were detected in exosomes, and elevated amounts of DR and CD63 were released into exosomes following expression of gB. It was therefore concluded that HSV-1 gB reduces the surface expression of DR by hijacking DR away from its normal transport route to the cell surface.

#### 3.2.3 Targeting CD74

CD74 is the invariant polypeptide chain involved in the transport of MHC-II molecules, and facilitates appropriate peptide loading to MHC-II complexes in the endolysosomal vesicles [65]. Its importance for antigen presentation is illustrated by the demonstration that knock-down of CD74 expression through siRNA technology can suppress the recognition by specific CD4 T cells [63]. It now appears that CD74 is a common target of viruses to modulate MHC-II antigen presentation. In a study of HSV-1 infection of B cells, Neumann *et al*. evaluated the total amount of CD74, DR, and DM at different times after HSV-1 infection, and showed that the expression of CD74 was dramatically reduced in infected cells from 18 h post-infection, falling to less than 15% at 64 h post-infection compared to uninfected cells, while the total cell levels of HLA-DR and -DM remained unaffected [79]. However, the level of SDS-stable MHC-II complexes, which represents the fraction of peptide-loaded MHC-II complexes was greatly reduced in the infected cells compared with uninfected cells. Together, these observations hint that the loss of CD74 expression leads to impaired peptide loading to the MHC-II complexes in the HSV-1-infected cells, with consequent impairment of MHC-II antigen presentation. 

Very recently we showed that EBV-encoded BZLF1 also targets CD74 to help the virus evade CD4 T cell recognition [63]. In functional CD4 T cell assays, BZLF1 was found to interfere with recognition by immune CD4 T cells. This impaired T cell recognition occurred in the absence of a reduction in the expression of surface MHC-II DR, but correlated with a marked downregulation of surface CD74 on the target cells. The surface CD74 downregulation by BZLF1 is mediated through an as yet unknown post-transcriptional mechanism that is distinct from previously reported effects of BZLF1 on the CIITA promoter. Interestingly, in addition to being a chaperone for MHC-II complex, CD74 also functions as a surface receptor for macrophage Migration Inhibitory Factor and enhances cell survival through transcriptional upregulation of Bcl-2 family members [81,82]. The immune-evasion function of BZLF1 therefore comes at a cost of induced toxicity. However, this toxicity can be overcome by expression of another E gene, BHRF1, which is a BCL-2 family homologue [63]. 

#### 3.2.4. Manipulation of T Cell Receptor (TCR) Recognition

The herpesviruses have evolved mechanisms to modulate MHC-II antigen presentation even after the MHC-II peptide complex reached the cell surface. The best studied example of this is the EBV-encoded BZLF2 protein (gp42) which also has a critical role in the binding and entry of EBV during infection of B cells by EBV [83,84]. In mixed lymphocyte culture assays of T cell function, gp42 can inhibit antigen-driven PBMC proliferation. Both the viral entry function and the immune-evasion function of gp42 derive from its ability to bind to the MHC-IIDR β-chain, which occurs in a domain that participates in the formation of peptide binding pockets [85]. By using MHC-II expressing human melanoma cells, a more detailed biochemistry study showed that EBV-encoded gp42 did not alter HLA-DR surface expression, nor its intracellular transport and maturation. But it was confirmed in a B cell model system that EBV encoded gp42 does reduce the ability of target cells to activate specific CD4 T Cells [86]. The underlying mechanism involves abolition of interactions between TCR on the CD4 T cell with MHC-II peptide complexes in the presence of gp42; crystal structure studies revealed that EBV gp42 sterically competes with TCR V-α domains [87]. There are two forms of gp42, a full-length type II membrane protein and a truncated soluble form, both of which can be detected in an EBV-producing Burkitt’s lymphoma cell line. Interestingly, this soluble form gp42 itself is sufficient to inhibit MHC-II antigen presentation in functional CD4 T cell assay [88]. This interference of TCR interaction with MHC-II complex by EBV gp42 is a novel mechanism to modulate MHC-II antigen presentation.

#### 3.2.5. Other Mechanisms of Manipulation of MHC-II Pathway

Notwithstanding the above mentioned specific immune evasion strategies of herpesviruses, there are other important but more general mechanisms of evasion from CD4 T cell responses. The first is global host protein synthesis shutoff following entry into lytic cycle, which is a feature of alpha- and gamma-herpesviruses. It can diminish antigen presentation on both the MHC-I and MHC-II antigen presentation pathways by limiting the availability of newly synthesised MHC molecules and their chaperones. The viral genes responsible are not homologous across the herpesvirus subfamilies. The first virus host shutoff (vhs) gene to be identified was UL41 of HSV-1 [89], which has endoribonuclease enzyme functions that effect host shutoff by decreasing the half-life of mRNAs [90,91]. Beta- and gamma-herpesviruses do not contain UL41 homologous genes, although gamma-herpesviruses encode enzymes that serve as vhs equivalents; for EBV, the relevant gene is BGLF5 [92] and for KSHV it is SOX [93]. Interestingly, the vhs proteins from gammaherpesviruses, are bifunctional proteins that were first identified as DNase/alkaline exonuclease enzymes [94,95] that are conserved across all herpesvirus families but which in gammaherpesviruses have evolved additional mRNase functions [96,97]. The degradation of mRNA induced by these viral proteins blocks the synthesis of MHC-II molecules, which is reflected by reduced levels of these MHC-II complexes on the cell surface. By using UL41 knockout HSV-1 virus, one study confirmed that UL41 can shut off the synthesis of MHC-II molecules and partly contribute to the downregulation of surface expression of MHC-II molecules in the whole virus context [98]. Although it has yet to be tested that this vhs protein or that of the gamma-herpesviruses can inhibit the recognition by CD4 T cells, in light of their effect on CD8 T cell recognition [95] it is reasonable to presume that the effect on MHC-II expression could lead to escape from CD4 T cell recognition. 

Another feature shared by many herpesviruses, including HCMV [99] and EBV [100] is that they encode viral homologues of interleukin 10 (vIL-10), which is known for its function of downregulating the cell surface MHC-II expression by preventing them reaching the cell surface [101]. Indeed, treating monocytes with recombinant CMV IL-10 can reduce the surface expression of MHC class II by about threefold compared with control mock-treated cells [102]. Recently, one study showed that infection of human primary endothelial cells with KSHV, which does not encode a vIL10 homologue, inhibits IFN-γ-induced expression of the MHC-II molecule at the transcriptional level; this effect was mediated by soluble factors, including cytokines released from the KSHV-infected endothelial cells [103]. 

## 4. Conclusion

Persistent lifelong infection by herpesviruses depends on the balance between host immune responses and viral immune evasion. On the one hand, herpesvirus infections elicit very strong T cell responses, on the other hand the herpesviruses have evolved mechanisms to interfere with MHC antigen presentation. Recent studies have demonstrated the capacity of herpesviruses to modulate MHC-II antigen presentation by targeting multiple points in the antigen processing pathway, and to impair recognition by virus-specific CD4 T cells. In addition, a number of herpesvirus gene products responsible for this modulation have been identified and characterized. Collectively these studies have enhanced our understanding of the normal biology of herpesvirus persistence, and have the potential to inform immunotherapeutic strategies to combat the pathogenic effects of these viral infections.
